# Acute cholecystitis in high risk surgical patients: percutaneous cholecystostomy versus laparoscopic cholecystectomy (CHOCOLATE trial): Study protocol for a randomized controlled trial

**DOI:** 10.1186/1745-6215-13-7

**Published:** 2012-01-12

**Authors:** Kirsten Kortram, Bert van Ramshorst, Thomas L Bollen, Marc GH Besselink, Dirk J Gouma, Tom Karsten, Philip M Kruyt, Grard AP Nieuwenhuijzen, Johannes C Kelder, Ellen Tromp, Djamila Boerma

**Affiliations:** 1 Dept. of Surgery, St. Antonius Hospital Nieuwegein; 2 Dept. of Radiology, St. Antonius Hospital Nieuwegein; 3 Dept. of Surgery, Academic Medical Centre Amsterdam; 4 Dept. of Surgery, Onze Lieve Vrouwe Gasthuis Amsterdam; 5 Dept. of Surgery, Hospital Gelderse Vallei Ede; 6 Dept. of Surgery, Catharina Hospital Eindhoven; 7 Dept. of Clinical Epidemiology. St. Antonius Hospital Nieuwegein

**Keywords:** Acute cholecystitis, laparoscopic cholecystectomy, percutaneous cholecystostomy, percutaneous drainage

## Abstract

**Background:**

Laparoscopic cholecystectomy in acute calculous cholecystitis in high risk patients can lead to significant morbidity and mortality. Percutaneous cholecystostomy may be an alternative treatment option but the current literature does not provide the surgical community with evidence based advice.

**Methods/Design:**

The CHOCOLATE trial is a randomised controlled, parallel-group, superiority multicenter trial. High risk patients, defined as APACHE-II score 7-14, with acute calculous cholecystitis will be randomised to laparoscopic cholecystectomy or percutaneous cholecystostomy. During a two year period 284 patients will be enrolled from 30 high volume teaching hospitals. The primary endpoint is a composite endpoint of major complications within three months following randomization and need for re-intervention and mortality during the follow-up period of one year. Secondary endpoints include all other complications, duration of hospital admission, difficulty of procedures and total costs.

**Discussion:**

The CHOCOLATE trial is designed to provide the surgical community with an evidence based guideline in the treatment of acute calculous cholecystitis in high risk patients.

**Trial Registration:**

Netherlands Trial Register (NTR): NTR2666

## Background

Acute calculous cholecystitis (ACC) is a frequently encountered disease in the general surgical practice. In young, otherwise healthy patients laparoscopic cholecystectomy (LC) is the treatment of choice [1]. In elderly patients with major comorbidity and seriously ill patients, especially those already admitted to an intensive care unit, percutaneous cholecystostomy (PC) seems preferable since acute LC in these patients can result in serious morbidity (up to 41%[2-7]) and mortality (up to 4.5%[2-6,8]). But there is a remaining subgroup of patients who can be defined as "high risk patients" based on their comorbidity or disease severity but do not fit in either of the two afore-mentioned categories.

Both LC and PC are often used in this subgroup of patients but clear selection criteria for either treatment are lacking and a number of questions regarding the safety and efficacy of PC remain.

In the Dutch guidelines for the treatment of gallstone disease [9] PC is indicated as a useful treatment option in patients deemed unfit for surgery but it is left open to discussion whether routine use of PC has additional value over antibiotic treatment in the therapy of ACC in the general population. Little evidence is provided to support this statement.

Most studies in the current literature addressing PC as a therapeutic option in ACC in high risk patients are retrospective with limited population sizes. Success rates are fairly high, but mortality rates of PC (range 4-12.7%[6]) are higher than those for LC. This could be attributed to selection bias as it is to be expected that those patients treated with PC were in a worse clinical condition than those treated with LC.

A systematic review conducted in 2007[6] analyzed the safety and efficacy of PC in elderly and critically ill patients. The review identified no clinical trials comparing PC with LC and the included studies were mostly retrospective and involved small patient populations. With a success rate of 91% in patients with confirmed ACC and a procedure related mortality of 0.4% results of PC seemed promising. The overall mortality was 12.7% and the overall complication rate was reported to be 6.2%. Complication rates were not included in more than half of the studies thus leading to an underestimation of the actual complication rate.

We performed a retrospective review of all patients undergoing PC for acute calculous cholecystitis between January 2009 and June 2010 [10]. A total of 27 patients were included with a median age of 83 years. PC was performed because of either comorbidity/age or duration of symptoms. The success rate was 92.6% (N = 25) but the complication rate was 22.2% (N = 6). The overall mortality rate was 14.8% (n = 4) and the procedure related mortality rate 3.7% (N = 1). With a mean follow-up of eight weeks three patients developed recurrent cholecystitis and four patients underwent an interval cholecystectomy.

Current literature and data from our own clinical experience fail to answer the question which therapy is the best option in ACC in high risk patients. At present both treatment strategies are used in this patient category and the preference and expertise of the responsible surgeon or the general opinion within the hospital usually determines the choice of treatment.

The CHOCOLATE trial is designed to assess which treatment modality is best suited for high risk patients with ACC, with the aim to prove superiority of the laparoscopic cholecystectomy.

## Methods

### Design

The CHOCOLATE trial is a randomised controlled, open, parallel, superiority multicenter study. Patients will be randomly allocated to undergo either laparoscopic cholecystectomy or percutaneous drainage.

The aim of this study is to test the hypothesis that laparoscopic cholecystectomy will lead to a reduction in morbidity and mortality compared to percutaneous drainage in high risk patients with acute calculous cholecystitis.

### Primary & Secondary endpoints

The primary endpoint is a composite endpoint of all major morbidity, re-intervention and mortality. Table 1 provides an overview of the definitions. Complications occurring during the first 30 days subsequent to randomisation and need for reintervention and mortality during the one year follow-up period will be compared.

**Table 1:** Definitions of the primary endpoint.

Endpoint	Definition
*Complications*	
Intra-abdominal abscess	Fever and/or elevated CRP/WBC and intra-abdominal fluid collection on CT-imaging or ultrasound.
Biliary injury	All injuries of the intra- and extrahepatic biliary ducts including leakage of the biliary tree*.
Bleeding	Drop in haemoglobin level requiring transfusion and/or reintervention.
Pneumonia	Coughing or dyspnoea, radiography with infiltrative abnormalities, elevated infection parameters and positive sputum culture.
Myocardial infarction	Symptomatic elevated cardiac enzymes and abnormalities on electrocardiography or cardiac ultrasound.
Cerebrovascular	(Temporary) loss of function of any body part or sense caused by cerebral ischemia or bleeding, proven on cerebral CT imaging.
Thrombo-embolic	Symptomatic deep venous thrombosis or pulmonary embolism, radiologically proven.
*Need for re-intervention*	Relaparoscopy, laparotomy, ERCP, intervention radiology, readmission
*Mortality*	

CRP - C-Reactive Protein; WBC - White Blood Cell count; CT - computed Tomography; ERCP - Endoscopic Retrograde Cholangiopancreaticography

* Type A: leakage of the minor hepatic ducts or cystic duct. Type B: leakage of common bile duct (CBD) without stricture - Type C: stricture of the CBD without bile leakage - Type D: complete transection of the CBD with or without resection of a part of it [11].

The secondary endpoints include all individual components of the primary endpoint and in addition all minor complications including wound infection, bleeding without need for transfusion or intervention and urinary tract infection, difficulty of cholecystectomy (as scored by VAS 1-10), total length of hospital stay, emergency room visits for related medical problems, re-admissions, duration of hospital and intensive care stay and total (direct and indirect) costs.

### Study Population

All patients presenting with ACC as defined according tot the Tokyo Guidelines [12] to one of the participating hospitals will be assessed for eligibility on presentation. If patients meet the inclusion criteria they will be stratified for hospital and randomised to undergo either LC or PC.

The in- and exclusion criteria are presented in table 2.

**Table 2:** In- and exclusion criteria for eligibility for participation in the CHOCOLATE trial.

Inclusion Criteria	Exclusion Criteria
- Age ≥ 18 - The diagnoses acute calculous cholecystitis defined according to the Tokyo Guidelines: A. Local signs of inflammation: (1) Murphy's sign, (2) Right upper quadrant mass/pain/tenderness B. Systemic signs of inflammation: (1) Fever, (2) elevated CRP, (3) elevated WBC count C. Imaging findings: imaging findings characteristic of acute cholecystitis Definite diagnosis: (1) One item in A and one item in B are positive (2) C confirms the diagnosis when acute cholecystitis is suspected clinically - APACHE-II score ≥ 7 AND ≤ 14 - Written informed consent	- Onset of symptoms > 7 days before first presentation - Already admitted to ICU on presentation - Pregnancy - APACHE-II score ≤ 6 OR ≥ 15 - Acalculous cholecystitis - Decompensated liver cirrhosis - Mental illness prohibiting informed consent

### Randomisation

**Figure 1:**
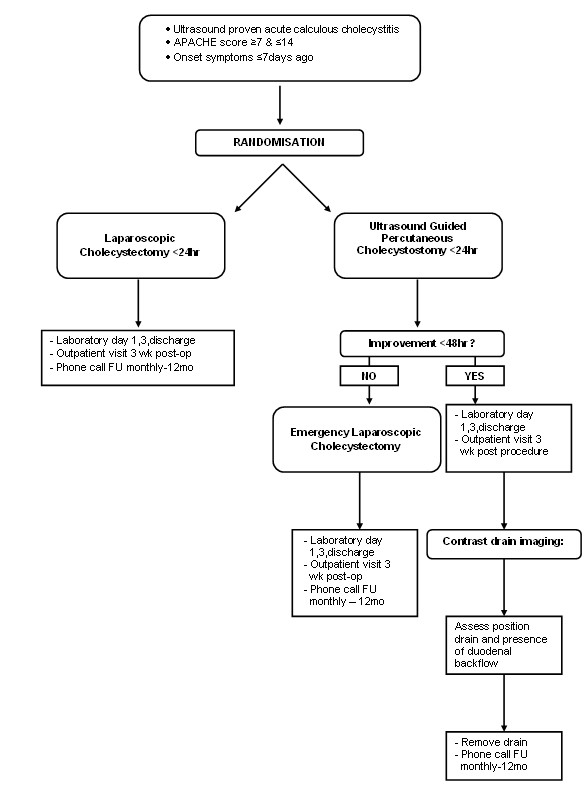
**Flowchart of study outline of included patients**.

Patients will be randomly assigned to group A (laparoscopic cholecystectomy) or group B (percutaneous drainage) as shown in the flowchart (Figure 1). Randomisation is done by the study coordinator or primary investigator using an online generator (ALEA 2.2, Academic Medical Centre Amsterdam, the Netherlands https://nl.tenalea.net/amc/ALEA/). Permuted-block randomisation with varying block sizes with a maximum block size of four patients is used to minimize the occurrence of chance imbalance and preserve unpredictability. The sequence of the different blocks is predetermined by an independent programmer and concealed to all investigators. The blocks are generated separately within the different study sites, so stratification according to hospital can be performed.

### Treatment protocol

All patients presenting with suspected ACC in the emergency department will undergo the standard laboratory work-up and an arterial blood gas analysis. An ultrasound of the abdomen will be performed to confirm the diagnosis. If the findings on ultrasound examination are inconclusive, a contrast enhanced CT-scan of the abdomen will be performed.

When patients are eligible for inclusion and informed consent is obtained randomisation will take place. The allocated treatment has to be performed within 24 hours after randomisation. Figure 1 demonstrates the study outline for included patients.

Group A: Laparoscopic cholecystectomy

LC will be performed by the four trocar technique with transsection of the cystic duct and cystic artery after reaching the critical view of safety as described in the Dutch Guidelines for the treatment of gallstone disease [9]. LC will be performed by a surgeon trained and experienced in laparoscopic surgery defined as > 100 laparoscopic procedures on a yearly basis.

Patients may receive prophylactic antibiotics according to the local hospital protocol.

Antibiotic therapy will not be routinely continued post-operatively unless the performing surgeon has strong indications to do so (such as (imminent) sepsis or hemodynamic instability). In these cases the primary investigator will be notified and the indications have to be well documented.

Group B: Percutaneous drainage

PC will be performed by either ultrasound- or CT-guided percutaneous drainage using an 8.5 French maclock drain. Since there is no consensus in the current literature [9,13] both the transhepatic and transperitoneal routes are allowed and which route is the safest in the individual patient is to be judged by the performing radiologist.

Successful PC is defined as resolution of symptoms and fever and normalization of C-Reactive protein and white blood count. Failure to thrive, defined as clinical deterioration, persisting fever, or increasing infection parameters < 48 hours despite accurate drain position and function will lead to LC.

Patients will be discharged with the drain in place. The drain will be left in situ during a period of three weeks, after which contrast-imaging of the drain will be performed prior to removal to assess whether the drain is still located in the gallbladder and whether there is a patent cystic duct. Patients undergoing PC will not be treated with antibiotics unless the patient is septic with (imminent) hemodynamic instability.

When patients in either group develop an infectious complication antibiotic therapy can be started. All events will be recorded.

### Data collection & follow-up

Each patient will receive an anonymous study number which will be used for the study case-record-forms (CRF) and the database.

On admission baseline patient characteristics including, gender, age, body-mass-index (BMI), comorbidity, previous abdominal surgery, APACHE-II score and duration of symptoms will be documented by the admitting physician or the (local) study coordinator.

The data regarding the procedure including difficulty, duration and complications, will be scored immediately after the procedure by the performing surgeon or radiologist.

During the admission a daily record of the patients including vital signs, laboratory data and complications will be kept.

After discharge the patient will be seen in the outpatient clinic by a surgeon who will complete a questionnaire regarding the patient's clinical condition. The following 11 months the study coordinator will perform the follow up by phone and fill out the CRF regarding complaints, readmissions and interventions.

All entered data will be checked for completion by the study coordinator every three months and missing data will be collected from the participating centers.

### Safety

A data safety monitoring board (DSMB) consisting of three independent members will monitor the safety and efficacy of the trial.

There will be a meeting every six months in which the DSMB will assess unblinded data and evaluate the current status of the trial.

### Adverse events

Adverse events are defined as any undesirable experience occurring to a subject during the study, whether or not considered related to the intervention (i.e. LC or PC).

All participating physicians will inform the study coordinator of all (serious) adverse events ((S)AE) immediately on occurrence.

The primary investigator or study coordinator will list all (S)AE and present these to the DSMB for every 30 randomized patients. All potential (S)AE will be reported to the Central Committee on Research involving Human Subjects ("Centrale Commissie Mensgebonden Onderzoek" [CCMO]) and the accredited medical ethical committee using the online module https://toetsingonline.ccmo.nl.

### Ethics

The CHOCOLATE trial is conducted in accordance with the declaration of Helsinki and the Dutch "Wet Medisch Wetenschappelijk Onderzoek met Mensen" (Medical Research Involving Human Subjects Act).

The study protocol was approved by the "Verenigde Commissies Mensgebonden Onderzoek" (United committees involving human research), the medical ethical committee of the trial coordinating centre at the St Antonius Hospital Nieuwegein on January 20^th ^2011. Secondary approval was obtained from all local ethics committees in the participating centres.

Patients will give oral as well as written informed consent prior to randomisation.

The CHOCOLATE trial is registered in the Dutch Trial Register with identification number NTR2666.

## Statistical aspects

### Sample size calculation

The rates for the primary endpoint: major morbidity, re-intervention and mortality for PC were 6.2, 13.1 and 12.7% respectively in the review and 22.2, 18.5 and 14.8% in our own series. We believe that the 6.2% complication rate in the review is an underestimation of the actual rate, but our own value of 22.2% was based on a retrospective, selected group of patients to undergo PC mostly because of their comorbidity. In these patients the complication rate may very well be higher than in the intended population of the CHOCOLATE trial. We therefore chose to combine both complication rates and use the mean value for our sample size calculation: 14.2%.

Morbidity rates for LC in high risk patients vary greatly in current literature and are mainly based on small, retrospective populations. Mortality rates are described to be around 4.5%. In our own series the complication rate was 13.6% and the mortality rate was 4.3%.

Our endpoint comprises major complications and mortality. These rates cannot be simply added to each other since mortality is in most case associated with major complications. We therefore chose to add intervention rates and complication rates and add up a hypothetical 1% for mortality to ensure that no cases positive for the primary endpoint were missed.

A decrease of the primary endpoint from 28.3% (PC group) to 14.6% (LC group) with power 80%, alpha two-sided 5%, Fisher exact, two proportions, 1:1 randomization can be demonstrated by randomizing 2 × 140 patients (PS Power and Sample Size Calculations, version 2.1.30, February 2003). With an expected loss to follow up of 1%, a total of 284 patients will have to be included in the trial.

### Descriptive statistics

Dichotomous data and counts will be presented in frequencies. Continuous data will be presented in means with a standard deviation or median value with a range.

### Analyses

All patients will be analysed according to the intention to treat approach. Baseline balance will be assessed based on the following patient factors: APACHE-II score, sex, age, duration of symptoms, previous abdominal surgery and previous biliary history. A significant difference (p-value < 0.05) in any of these factors between the two treatment groups will be considered to be an imbalance, the most important factors being APACHE-II score and age. Although we do not expect this to occur, results within the treatment groups will be stratified according to APACHE-II score and age to assure reliable results.

For nominal data the Chi-Square test will be used. For continuous data and counts the independent sample t-test or one-way ANOVA will be used.

In the interim analysis (after one year) the occurrence of the primary endpoint will be compared between the two treatment groups. After the one year follow up of the last patient is completed primary as well as secondary outcomes will be analyzed and compared.

Results will be presented as odds ratios with a corresponding 95% confidence interval. A two-tailed P < 0.05 is considered statistically significant.

### Premature termination of the study

The results of the interim analysis performed after the first year of inclusion will be evaluated by the independent DSMB. The primary endpoint will be monitored for benefit or harm using a restricted procedure (Whitehead, 1997), designed according to the sample size characteristics as described in the protocol. The Peto approach will be followed meaning that the study will only be stopped for beneficial effects in case of a p-value < 0.001.

Safety monitoring will be performed specifically for mortality with a two-sided type I error (α) of 0.05. A relative risk of ≥ 2 will be considered reason to advise to terminate the trial.

The trial will not be stopped for futility, the reason being that this is the first randomized controlled trial on this subject and treatment policy will be based on this trial.

### Feasibility

The CHOCOLATE trial was registered in the Dutch Trial Register on December 27^th ^2010. Approval from the medical ethical committee was obtained on January 20^th ^2011 and the first patient was randomised on February 23^rd ^2011. Presently local approval by the ethical committees has been obtained in 15 participating centres and six more centres have already been invited tot participate in the study. After one year the inclusion rate will be assessed. If Accrual is too slow, additional centres will be invited to participate.

## Discussion

The treatment of ACC in high risk patients is a much debated subject in the surgical community and evidence based guidelines are lacking. Current literature does not provide an answer to the essential question which is the better therapy: LC or PC? The CHOCOLATE trial is designed with the aim to determine superiority of the LC.

The best design for a therapeutic trial is a randomised placebo-controlled, double-blind study but considering the different interventions used in the CHOCOLATE trial design is not feasible and therefore a randomised open comparative design was chosen.

It is generally accepted that in young, otherwise healthy patients LC is the treatment of choice for ACC [1]. In elderly patients with major comorbidity or seriously ill patients PC seems preferable. A subgroup of patients remains which on the basis of co-morbidity or disease severity can be defined as higher risk patients but cannot be assigned to either of the mentioned categories. In this subgroup of patients it remains unclear from which treatment option they will benefit the most with the least risks. The definition of this subgroup was set by a multicentre, multidisciplinary expert panel. A number of imaginary case scenarios including age, comorbidity, vital signs and laboratory findings on presentation were presented to the panel. Subsequently the panel was asked which therapy (LC or PC) they would choose for their patient and whether either one would be contra-indicated. Multiple scoring systems were evaluated by the panel and the APACHE-II score proved the best system to discriminate between patient groups. There was consensus that patients scoring < 7 should undergo emergency LC, and patients scoring > 14 were to have PC. In patients scoring between 7 and 14, opinions differed and no consensus was reached regarding which treatment was better, resulting in the inclusion criterion of the APACHE-II score of 7-14.

We incorporated the condition that all LC patients need to be operated on by or under supervision of a laparoscopically experienced surgeon to ensure that all patients receive the best possible treatment. A previous study demonstrated significantly higher conversion rates in LC for ACC when the surgery was performed by non-laparoscopic surgeons [14]. Standardizing this aspect of this treatment arm will make for a homogenous patient group and prevent confounding based on the performing surgeon.

With these medical, technical and ethical considerations in mind we created the trial protocol according to this described design.

## Conclusion

The CHOCOLATE trial is a randomised controlled multicenter study comparing laparoscopic cholecystectomy with percutaneous drainage to determine the best treatment for acute calculous cholecystitis in high risk patients.

## Trial status

Approval of the study protocol from the central medical ethical committee as well as all local ethics committees has been obtained. Patients are being included in the trial in all participating centres. Currently 34 patients are enrolled in the CHOCOLATE trial.

## List of abbreviations

ACC: Acute Calculous Cholecystitis; AE: Adverse Event; ANOVA: Analysis of Variance; APACHE: Acute Physiology and Chronic Health Evaluation; CRF: Case Record Form; CRP: C-Reactive Protein; CT: Computed Tomography; DSMB: Data Safety Monitoring Board; ERCP: Endoscopic Retrograde Cholangiopancreaticography; LC: Laparoscopic Cholecystectomy; PC: Percutaneous Drainage; SAE: Serious Adverse Event; VAS: Visual Analogue Scale; WBC: White Blood Cell.

## Competing interests

The authors KK, BVR, TB, MB, DG, TK, PHK, GN, JK, ET and DB declare that they have no competing interests.

## Authors' contributions

KK drafted the manuscript

BVR and DB co-authored the writing of the manuscript

KK, BVR, MB, DG, TK, PHK, GN, JK, ET and DB participated in the design of the study

KK and MB performed the sample size calculations

All authors edited the manuscript and read and approved the final manuscript.

## Funding

There are no sources of funding for the authors of this manuscript.
